# Clinical and prognostic implications of CD47 and PD-L1 expression in surgically resected small-cell lung cancer

**DOI:** 10.1016/j.esmoop.2022.100631

**Published:** 2022-11-16

**Authors:** C. Lang, A. Lantos, Z. Megyesfalvi, F. Egger, M.A. Hoda, B. Mosleh, T. Klikovits, F. Oberndorfer, G. Timelthaler, B. Ferencz, J. Fillinger, A. Schwendenwein, A.S. Querner, K. Boettiger, F. Renyi-Vamos, K. Hoetzenecker, V. Laszlo, K. Schelch, B. Dome

**Affiliations:** 1 Department of Thoracic Surgery, Medical University of Vienna, Vienna, Austria; 2 National Korányi Institute of Pulmonology, Budapest; 3 Department of Thoracic Surgery, National Institute of Oncology, Semmelweis University, Budapest, Hungary; 4 Department of Thoracic Surgery, Clinic Floridsdorf, Vienna; 5 Department of Pathology, Medical University of Vienna, Vienna; 6 Center for Cancer Research, Medical University of Vienna, Vienna, Austria; 7 Department of Translational Medicine, Lund University, Lund, Sweden

**Keywords:** small-cell lung cancer, novel therapeutic targets, emerging prognostic factors, immune-checkpoint molecules

## Abstract

**Background:**

Pharmacological inhibition of the immune-checkpoint molecule CD47 has shown promising results in preclinical small-cell lung cancer (SCLC) models, whereas anti-programmed death-ligand 1 (PD-L1) inhibitors have been recently implemented in the standard of care of advanced-stage SCLC patients. Nevertheless, the expression pattern, clinical relevance and prognostic implication of both CD47 and PD-L1 are rather controversial in surgically treated SCLC patients.

**Materials and methods:**

In total, 104 Caucasian SCLC patients from two Central European thoracic centers were included in this study. CD47 and PD-L1 expression as well as the expression of the four major SCLC molecular subtype markers (ASCL1, NEUROD1, YAP1 and POU2F3) were measured by immunohistochemistry. Expression levels were independently evaluated and statistically correlated with clinicopathological data and survival.

**Results:**

Positive CD47 and PD-L1 expressions were seen in 84.6% and 9.6% of the samples, respectively. Meanwhile, the tumor-associated stroma was positive for PD-L1 in 59.6% of the cases. Stromal PD-L1 expression correlated with longer overall survival (OS) (versus PD-L1-negative stroma; median OS was 42 versus 14 months, respectively, *P* = 0.003) and was confirmed as an independent predictor of favorable outcome upon multivariate analysis (hazard ratio 0.530, 95% confidence interval 0.298-0.943, *P* = 0.031). Notably, neither CD47 nor PD-L1 presence was related to a distinct molecular SCLC subtype.

**Conclusion:**

CD47 shows a remarkably high expression while tumoral PD-L1 expression is generally low in surgically treated SCLC. Importantly, stromal PD-L1 expression may indicate a favorable clinical outcome and serve as a novel prognostic factor in these patients. Additional studies are warranted to further investigate the clinical impact of CD47 and PD-L1 expression in SCLC.

## Highlights

• CD47 and PD-L1 are promising therapeutic targets in SCLC.
• This study investigated CD47 and PD-L1 expressions in an international cohort of surgically resected SCLC specimens.
• CD47 is highly expressed while PD-L1 exhibits weak expression in surgically resected SCLC tumors.
• PD-L1 positivity in the tumor stroma robustly indicates favorable clinical outcome.
• Adjuvant chemotherapy was identified as the most important clinicopathological factor for improved survival.

## Introduction

Small-cell lung cancer (SCLC) is one of the most aggressive types of lung cancer (LC) and accounts for ∼15% of all LC cases worldwide. Historically, SCLC has been regarded as a cancer of neuroendocrine origin and is clinically characterized by rapid tumor progression and early metastatic dissemination. Although surgical resection has been shown to potentially offer favorable long-term outcomes, it is rarely carried out in SCLC^1^^,^^2^ since the vast majority of patients are being diagnosed with metastatic disease when local resection is not indicated anymore.^3^ Accordingly, there is a need for novel therapeutic concepts as clinical outcomes in SCLC remain extraordinarily poor with 5-year survival rates below 10%.^4^

Recent advancements in the field of SCLC research might provide hope for improved outcomes in this recalcitrant type of disease.^5^^,^^6^ Firstly, nationwide screening programs have been shown to effectively increase the number of limited-stage LC cases and to significantly reduce LC-associated mortality.^7^^,^^8^ Consequently, the overall number of potentially resectable patients is expected to increase in the upcoming decades. It is evident that there is a need for novel therapeutically tractable molecules and prognostic biomarkers for surgically managed patients.^9^ Secondly, evidence that SCLC may represent a far more heterogeneous malignancy than previously assumed is also growing.^10^ Indeed, a new model of SCLC nomenclature defined by the differential expression of four key-transcription regulators (ASCL1, NEUROD1, YAP1 and POU2F3) has just been recently presented and validated in surgically resected samples.^11^^,^^12^ Importantly, these distinct molecular subsets have major differences in cellular origin, neuroendocrine differentiation and growth properties.^10^^,^^13^ In addition, our group previously showed that the molecular subtypes of SCLC are also linked with different prognostic outcomes since high ASCL1 expression is associated with poor survival, whereas high POU2F3 expression is linked with improved prognosis.^12^

A significant number of targetable molecules have also been identified in preclinical studies and clinical trials recently.^10^ Most importantly, immunotherapeutic concepts have been discussed as especially promising for future perspectives in SCLC.^14^ Indeed, representing the most significant therapeutic novelties for decades, immunotherapeutic targeting of anti-programmed death-ligand 1 (PD-L1) has been recently included into the treatment guidelines for extensive-stage SCLC patients.^15^^,^^16^ Moreover, immune-checkpoint-mediated blockage of CD47, a ‘don’t-eat-me’ cell-surface signal for macrophages, has been shown to effectively suppress SCLC tumor growth *in vitro* and *in vivo.*^17^ This is of clinical importance for SCLC as clinical trials targeting CD47 by systemic immunotherapy are already enrolling patients with different solid and hematologic malignancies.^18^

Because only a few studies with small cohorts have aimed to examine the distribution and prognostic relevance of PD-L1 and CD47 in SCLC to date, the findings are preliminary and controversial and thus warrant further investigations.^19^^,^^20^ Accordingly, in the current study we aimed to evaluate (i) the expression pattern of CD47 and PD-L1, (ii) the relation between CD47/PD-L1 expression and recently discussed major molecular SCLC subtypes and (iii) the prognostic impact of CD47 and PD-L1 expression in a large international cohort of surgically treated Caucasian SCLC patients.

## Materials and methods

### Study design and clinical data

This study included histologically confirmed SCLC patients who underwent diagnostic or therapeutic surgical resection at two Central European high-volume thoracic surgical centers (Department of Thoracic Surgery, Medical University of Vienna, Vienna, Austria and Department of Thoracic Surgery, National Koranyi Institute of Pulmonology, Budapest, Hungary) between January 2000 and December 2019. Clinical and follow-up data were retrospectively extracted from institutional medical records or obtained directly from the Central Statistical Office. Wedge resection and segmentectomy were defined as sublobar resection, whereas lobectomy or pneumonectomy as lobar resection. Staging of the patients was retrospectively carried out using the data from histological findings and medical records in accordance with the eighth edition of the TNM (tumor–node–metastasis) classification for LC by the International Association for the Study of Lung Cancer.^21^^,^^22^ Recurrent disease was termed as evidence of recurrence in either mediastinal or hilar lymph nodes or in the ipsilateral lung. Evidence of any other recurrences was classified as distant organ metastases.

If recurrence or metastasis was suspected, further clinical evaluation [e.g. bronchoscopical/transthoracic biopsy of suspect lesion, positron emission tomography–computed tomography (CT), magnetic resonance imaging] was carried out according to the individual scenario. Overall survival (OS) was defined as the time of surgery until the date of the last available follow-up or death in months. Disease-free survival (DFS) was calculated as the time of surgery until the evidence of metastases or recurrent disease in months.

According to institutional protocols, routine oncological follow-up was carried out for all included patients. Notably, these follow-ups comprised regular (i.e. every 3 months in the first post-operative year and then every 6-12 months) blood tests, X-rays and/or thoracic CT scans of the chest. Distinct patients received tumor-specific adjuvant treatment based on the decision of a multidisciplinary tumor board consisting of board-certified medical, radiation and surgical oncologists in accordance with the contemporary National Comprehensive Cancer Network (NCCN) guidelines.^23^ Notably, there were no relevant differences across the two host institutions. Adjuvant chemotherapy consisted of a platinum agent (cisplatin or carboplatin) combined with etoposide. In rare cases, irradiation was also applied.

### Immunohistochemistry and evaluation

Surgically resected, formalin-fixed, paraffin-embedded primary tumor and lymph node specimens were obtained from the pathological departments of the participating institutions. Briefly, tissue specimens were freshly cut in 4-μm-thick sections and expression levels of CD47, PD-L1, ASCL1, NEUROD1, YAP1 and POU2F3 were examined by immunohistochemistry (IHC) according to the manufacturers’ protocol. The following antibodies and dilutions were applied: anti-CD47 (Sigma Aldrich, Burlington, MA, HPA044659, 1 : 100), anti-PD-L1 (Cell Signaling Technology, Leiden, The Netherlands, E1L3N/#13684, 1 : 100), anti-ASCL1 (BD Bioscience, San José, CA, #556604, 1 : 50), anti-NEUROD1 (Abcam, Cambridge, UK, #ab213725, 1 : 100), anti-YAP1 (Cell Signaling Technology, Leiden, The Netherlands, #4912, 1 : 200) and anti-POU2F3 (Santa Cruz Biotechnology, Dallas, TX, #sc-293402, 1 : 100). Sections were stained overnight at 4°C and then incubated with a secondary antibody for 1 h at room temperature. Finally, visualization of the expression levels was achieved with Liquid DAB+ Substrate Chromogen System (Dako, K3468) and sections were counterstained with hematoxylin. Antibodies and staining kits were both validated by using appropriate positive and negative tissue controls.

IHC staining results were independently evaluated in a double-blinded fashion by two experienced pathologists specialized in pulmonary malignancies. If there were significant differences between ratings, the section was discussed and re-evaluated. CD47 expression was scored as negative (absent or <10% positivity of all tumor cells) or positive (≥10% positivity of all tumor cells) considering membranous and/or cytoplasmic signals. For PD-L1, tumoral and stromal expression were both evaluated and defined as either negative (absent or <1% of tumor or stromal cells) or positive (≥10% of tumor or stromal cells). For ASCL1, NEUROD1, YAP1 and POU2F3, the previously defined cut-off values were used.^12^

### Statistical analysis

Collected and generated data were evaluated with GraphPad Prism 8 (GraphPad Software, Inc., San Diego, CA), Microsoft Excel Version 16.48 (Microsoft Corporation, Redmond, WA) and SPSS Statistics Version 27.0 (IBM Corp., Armonk, NY). The chi-square test was applied for the comparison of binominal variables. If the expected frequency was below 5, Fisher’s exact test was used. In order to identify risk factors for OS and DFS, all clinicopathological data and staining results were included in a univariate analysis using the Cox regression model and presented as hazard ratios (HRs) including corresponding 95% confidence intervals (CIs). Risk factors with significant results in univariate analysis were consecutively included for multivariate analysis to test for robustness and independency. For survival curve estimation, the Kaplan–Meier method together with the log-rank test was applied to assess significant differences in OS and DFS. All tests were calculated in a two-sided manner and *P* values of <0.05 were defined as statistically significant.

### Ethical statement

This study has been approved by the responsible ethics committees (Medical University of Vienna EK#: 2196/2019 and National Koranyi Institute of Pulmonology ETT-TUKEB 23636–2/2018, 23 636/10/2018/EÜIG) and was conducted with regard to the Declaration of Helsinki and the institutional Good Scientific Practice guidelines.

## Results

### Characterization of the study cohort

Altogether, 104 SCLC patients undergoing surgical resection between January 2000 and December 2019 were included. The median age at the time of surgery was 64 years (range 41-83 years). Forty-nine patients (47.1%) were female and 87 patients (83.7%) had tobacco exposure according to the medical records. The most common comorbidities were arterial hypertension (51%), chronic obstructive pulmonary disease (COPD) (41.3%) and diabetes mellitus (16.3%). Regarding the type of surgery, the most common procedures were lobectomy (51%), segmentectomy (13.5%), wedge resection (11.5%) and pneumonectomy (10.6%). Fifty-seven patients (54.8%) had limited-stage disease (stage I + II) and 62 patients (59.6%) received adjuvant chemotherapy according to local protocols. Clinicopathological data of the study population with regard to CD47 and PD-L1 expression are shown in Table 1 and Supplementary Table S1, available at https://doi.org/10.1016/j.esmoop.2022.100631.

**Table 1:** Summary of the main clinicopathological characteristics of the study cohort comprising 104 patients undergoing surgical resection for small-cell lung cancer (SCLC)

Clinicopathological characteristics	Total (*n* = 104) *n* (%)
Age (years, median, range)	63.7 (41.1-83.0)
<65	56 (53.8)
≥65	47 (45.2)
Not available (N/A)	1 (0.9)
Gender	
Female	49 (47.1)
Male	54 (51.9)
N/A	1 (0.9)
Smoking status (cigarette smoking only)	
Never smoker (less than 100 cigarettes / lifetime)	12 (11.5)
Former smoker (at least 100 cigarettes / lifetime but quitted smoking prior to surgery)	33 (31.7)
Current smoker (at least 100 cigarettes / lifetime and actively smoking at the time of surgery)	54 (51.9)
Unknown	5 (4.8)
Most common comorbidities	
Chronic obstructive pulmonary disease (COPD)	43 (41.3)
Hypertension	53 (51.0)
Diabetes mellitus	17 (16.3)
Surgery type	
Wedge resection	12 (11.5)
Segmentectomy	14 (13.5)
Lobectomy	53 (51.0)
Pneumonectomy	11 (10.6)
Unspecified	14 (13.5)
Stage	
I	39 (37.5)
II	18 (17.3)
≥III	31 (29.8)
N/A	16 (15.4)
Lymph node status	
N0	44 (42.3)
N1	23 (22.1)
N2	17 (16.3)
N/A	20 (19.2)
Tumor size	
T1	42 (40.4)
T2	22 (21.1)
T3	11 (10.6)
T4	12 (11.5)
N/A	17 (16.3)
Adjuvant chemotherapy	
Yes	62 (59.6)
No	24 (23.1)
N/A	18 (17.3)

### High presence of CD47 in surgically resected SCLC

**Figure 1:**
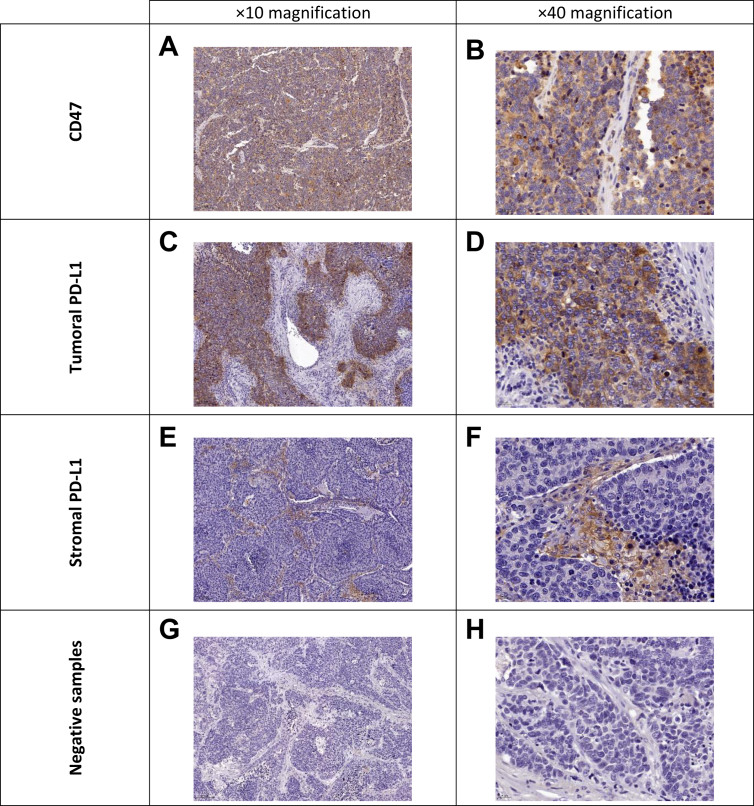
**Representative images of CD47 and PD-L1 expressions detected by IHC in surgically resected SCLC specimens.** CD47 was solely expressed by tumor cells (A, B) whereas PD-L1 was both detected in tumoral (C, D) and stromal (E, F) areas. Representative specimens without positivity for CD47 or PD-L1 are shown in G and H. IHC, immunohistochemistry; PD-L1, programmed death-ligand 1; SCLC, small-cell lung cancer.

Positive CD47 expression was detected in 88 out of 100 (84.6%) patients. However, CD47 expression did not correlate with clinicopathological patient characteristics. There was additionally no association between the expression of CD47 and the molecular SCLC subtypes within the study cohort (Supplementary Table S1, available at https://doi.org/10.1016/j.esmoop.2022.100631). Notably, four specimens had to be excluded due to inadequate staining quality. Representative samples of specimens stained for CD47 are shown in Figure 1A and B.

### Weak tumoral and moderate stromal PD-L1 presence in surgically resected SCLC

In contrast to CD47, positive tumoral PD-L1 expression (t-PD-L1^pos^) was less present in 10 out of 102 (9.6%) patients. Interestingly, there was a significantly higher number of t-PD-L1^pos^ patients in limited-stage disease (70% of t-PD-L1^pos^ were stage I + II, *P* = 0.0472, Supplementary Table S1, available at https://doi.org/10.1016/j.esmoop.2022.100631). Tumoral PD-L1 status did not significantly associate with any of the stained molecular SCLC subtypes.

When assessing the stromal area, we identified 62 patients (59.6% of the study cohort) showing positivity (>1%) for stromal PD-L1 expression (st-PD-L1^pos^). Interestingly, st-PD-L1^pos^ patients were more likely grouped into the N0 stage compared to st-PD-L1^neg^ patients (53.2% versus 25%, *P* = 0.024, Supplementary Table S1, available at https://doi.org/10.1016/j.esmoop.2022.100631). Stromal PD-L1 status did not vary between molecular SCLC subtypes. Of note, two samples stained for PD-L1 were excluded from further analysis due to poor staining quality. Representative samples showing various tumoral and stromal PD-L1 status are found in Figure 1C-F.

### Impact of clinicopathological characteristics on clinical outcome

**Figure 2:**
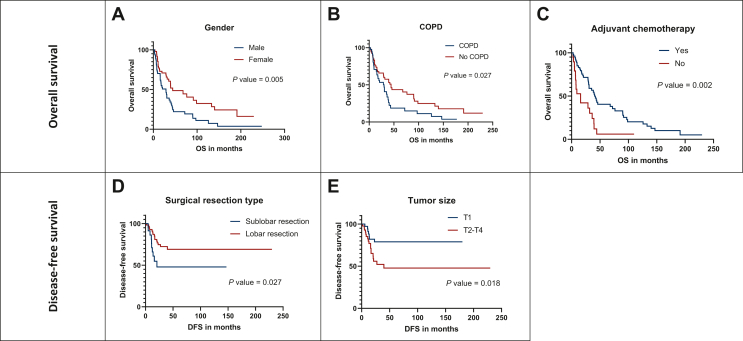
**Clinicopathological characteristics of the study cohort that significantly associate with clinical outcome following surgical resection for SCLC.** Male patients (A) and patients with COPD (B) had significantly worse OS while patients receiving adjuvant chemotherapy had clearly longer OS (C). Patients undergoing sublobar resection (D) or having tumor >T2 (E) had significantly impaired DFS. COPD, chronic obstructive pulmonary disease; DFS, disease-free survival; OS, overall survival; SCLC, small-cell lung cancer.

Next, we aimed to evaluate the impact of clinicopathological characteristics on OS and DFS after receiving surgical resection for SCLC. Interestingly, male patients (versus female, 20 versus 44 months, *P* = 0.0053) and patients with COPD (versus patients without COPD, 30 versus 44 months, *P* = 0.0266) had significantly worse OS while patients receiving adjuvant chemotherapy (versus those without adjuvant chemotherapy, 41 versus 16 months, *P* = 0.0015) had significantly longer OS (Figure 2A-C). Accordingly, univariate analysis confirmed gender (male, HR 1.954, 95% CI 1.209-3.158, *P* = 0.006) and COPD (HR 1.712, 95% CI 1.059-2.770, *P* = 0.028) as significant risk factors of impaired OS whereas adjuvant chemotherapy (HR 0.399, 95% CI 0.221-0.717, *P* = 0.002) was associated with longer OS following surgical resection in SCLC. After application of the multivariate analysis, adjuvant chemotherapy remained the only independent predictor of favorable OS (HR 0.399, 95% CI 0.211-0.755, *P* = 0.005, Table 2).

**Table 2:** **Univariate and multivariate analyses for identification and evaluation of risk factors affecting overall survival (OS) following surgical resection in SCL**C

	Univariate analysis			Multivariate analysis		
OS	HR	95% CI	*P* value	HR	95% CI	*P* value
Age (years): ≥65 versus <65	1.079	0.671-1.735	0.755			
Gender: male versus female	1.954	1.209-3.158	0.006^a^	1.718	0.904-3.262	0.098
Smoking status (cigarette smoking only)						
Smoker (at least 100 cigarettes / lifetime) versus never smoker (less than 100 cigarettes / lifetime)	0.924	0.457-1.866	0.825			
Current smoker (at least 100 cigarettes / lifetime and actively smoking at the time of surgery) versus non-current smoker (less than 100 cigarettes / lifetime or at least 100 cigarettes / lifetime but quitted smoking prior to surgery)	1.088	0.674-1.757	0.729			
Chronic obstructive pulmonary disease	1.712	1.059-2.770	0.028^a^	1.652	0.870-3.139	0.125
Hypertension	1.277	0.789-2.066	0.320			
Diabetes mellitus	1.353	0.706-2.593	0.362			
Surgery type: sublobar versus lobar resection	1.567	0.917-2.676	0.100			
Pathologic stage: early stage (=stage I + II) versus advanced stage (≥stage III)	0.879	0.523-1.478	0.628			
Lymph node status: N0 versus ≥N1	0.744	0.443-1.250	0.264			
Tumor size: T1 versus ≥T2	0.695	0.416-1.159	0.163			
Lesion pre-op visible on bronchoscopy	1.331	0.684-2.588	0.399			
Adjuvant chemotherapy	0.399	0.221-0.717	0.002^a^	0.399	0.211-0.755	0.005^a^
CD47: ≥10% versus <10%	1.181	0.562-2.482	0.661			
Tumoral PD-L1: positive versus negative	0.407	0.128-1.299	0.129			
Stromal PD-L1: positive versus negative	0.487	0.302-0.785	0.003^a^	0.530	0.298-0.943	0.031^a^

CI, confidence interval; HR, hazard ratio; PD-L1, programmed death-ligand 1; SCLC, small-cell lung cancer.

a Statistically significant.

Regarding DFS, patients receiving sublobar resection (versus lobar resection, 14 versus 29 months, *P* = 0.027) and those with T1 tumors (versus ≥T2 tumors, 30 versus 18 months, *P* = 0.018) were identified as having prognostic clinicopathological conditions (Figure 2D and E). Notably, sublobar resection was confirmed as a risk factor of worse DFS through univariate (HR 2.424, 95% CI 1.077-5.456, *P* = 0.032) and moreover, as an independent prognosticator on multivariate analysis (HR 3.274, 95% CI 1.407-7.618, *P* = 0.006). Similarly, tumor size also remained as a significant and independent clinical predictor of improved DFS in the univariate (T1 versus ≥T2, HR 0.362, 95% CI 0.151-0.869, *P* = 0.023) and multivariate analysis (T1 versus ≥T2, HR 0.351, 95% CI 0.144-0.858, *P* = 0.022) (Table 3).

### Prognostic implications of CD47 and PD-L1 expression

We lastly aimed to assess the prognostic value of CD47 and PD-L1 status for the clinical outcome regarding post-operative OS and DFS of patients.

**Figure 3:**
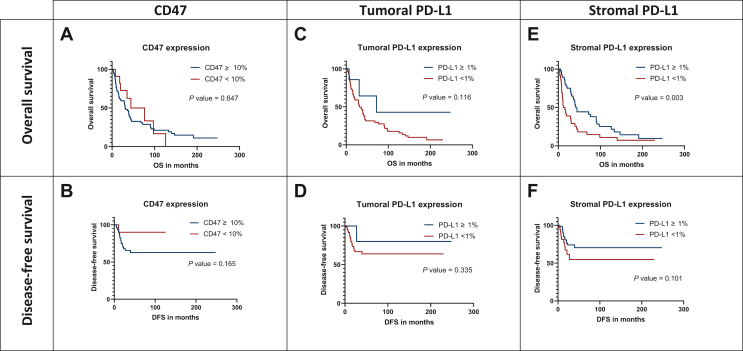
**Kaplan–Meier survival curves evaluating the prognostic value of CD47 and PD-L1 status in surgically resected SCLC.** Patients with increased CD47 presence had a tendency toward shorter OS and DFS (A, B). Positive tumoral PD-L1 status was associated with longer OS and DFS (C, D). A clear and significantly strong association with favorable outcome for patients with positive stromal PD-L1 was detected (E, F). DFS, disease-free survival; OS, overall survival; PD-L1, programmed death-ligand 1; SCLC, small-cell lung cancer.

Positive CD47 status correlated with shortened OS (31 versus 44 months, *P* = 0.647) and DFS (21 versus 35 months, *P* = 0.165) (Figure 3A and B). In accordance, univariate analysis calculated a slightly increased risk of shorter OS (HR 1.181, 95% CI 0.562-2.482, *P* = 0.661) and a clearly heightened risk of shorter DFS (HR 3.745, 95% CI 0.505-27.798, *P* = 0.197) in patients with CD47-expressing tumors (Tables 2 and 3).

**Table 3:** Univariate and multivariate analyses for identification and evaluation of risk factors affecting disease-free survival (DFS) following surgical resection in SCLC

	Univariate analysis			Multivariate analysis		
DFS	HR	95% CI	*P* value	HR	95% CI	*P* value
Age (years): ≥65 versus <65	0.901	0.409-1.986	0.796			
Gender: male versus female	1.772	0.796-3.947	0.161			
Smoking status (cigarette smoking only)						
Smoker (at least 100 cigarettes / lifetime) versus never smoker (less than 100 cigarettes / lifetime)	1.056	0.315-3.544	0.930			
Current smoker (at least 100 cigarettes / lifetime and actively smoking at the time of surgery) versus non-current smoker (less than 100 cigarettes / lifetime or at least 100 cigarettes / lifetime but quitted smoking prior to surgery)	1.607	0.701-3.682	0.262			
Chronic obstructive pulmonary disease	1.350	0.614-2.968	0.456			
Hypertension	1.004	0.458-2.202	0.991			
Diabetes mellitus	0.500	0.118-2.123	0.348			
Surgery type: sublobar versus lobar resection	2.424	1.077-5.456	0.032^a^	3.274	1.407-7.618	0.006^a^
Pathologic stage: early stage (=stage I + II) versus advanced stage (≥stage III)	0.849	0.375-1.924	0.696			
Lymph node status: N0 versus ≥N1	0.494	0.207-1.177	0.111			
Tumor size: T1 versus ≥T2	0.362	0.151-0.869	0.023^a^	0.351	0.144-0.858	0.022^a^
Lesion pre-op visible on BSC	1.725	0.547-5.443	0.352			
Adjuvant chemotherapy	0.997	0.373-2.664	0.996			
CD47: ≥10% versus <10%	3.745	0.505-27.798	0.197			
Tumoral PD-L1: positive versus negative	0.388	0.052-2.870	0.354			
Stromal PD-L1: positive versus negative	0.521	0.236-1.153	0.108			

CI, confidence interval; HR, hazard ratio; PD-L1, programmed death-ligand 1; SCLC, small-cell lung cancer.

a Statistically significant.

Regarding PD-L1 expression, patients with positive tumoral PD-L1 status showed a tendency toward longer OS (72 versus 32 months, *P* = 0.116) and DFS (30 versus 20 months, *P* = 0.335) (Figure 3C and D). Moreover, univariate analysis calculated a lower risk of events regarding OS (HR 0.407, 95% CI 0.128-1.299, *P* = 0.129) and DFS (HR 0.388, 95% CI 0.052-2.870, *P* = 0.354) (Tables 2 and 3).

Interestingly, patients with positive stromal PD-L1 expression had significantly longer OS (42 versus 14 months, *P* = 0.003) and a tendency toward longer DFS (30 versus 12 months, *P* = 0.101) (Figure 3E and F). Importantly, stromal PD-L1 expression remained a strong and independent predictor of favorable OS both in the univariate (HR 0.487, 95% CI 0.302-0.785, *P* = 0.003) and multivariate Cox regression analyses (HR 0.530, 95% CI 0.298-0.943, *P* = 0.031) (Tables 2 and 3). Regarding DFS, the univariate (HR 0.521, 95% CI 0.236-1.153, *P* = 0.108) analysis again confirmed positive stromal PD-L1 status as a predictor of a favorable outcome but did not reach the level of statistical significance.

## Discussion

In this study, we aimed to investigate the presence and prognostic value of the immune-checkpoint molecules CD47 and PD-L1 in a comparatively large international cohort of surgically resected SCLC patients. We identified distinct clinicopathological characteristics and the therapeutic approaches that significantly affect the outcomes of surgically resected SCLC patients. Moreover, we showed that CD47 is strongly expressed while PD-L1 presence is generally low in SCLC tissue. Ultimately, we demonstrated that stromal PD-L1 expression significantly correlates with improved outcome and represents an independent prognostic biomarker in resected SCLC.

SCLC represents one of the most aggressive types of LC since clinically it is characterized by extraordinarily poor survival rates. Although the overall clinical outcome of SCLC patients did not undergo significant improvements over the last decades, distinct demographics and treatment modalities associate with remarkable survival differences.^24^, ^25^, ^26^ Indeed, we identified the application of adjuvant chemotherapy as one of the strongest predictors of longer OS in our study. Moreover, we also found a clear association between increasing tumor size (≥T2) and less-radical surgical resection (sublobar versus lobar resection), and worse DFS. Accordingly, our findings confirm comparable results from previous reports and should be considered when applying surgery in SCLC patients.^27^, ^28^, ^29^, ^30^

Regarding the relevance of CD47 expression in SCLC, we detected positive expression in 88% (88/100) of all evaluated patients. Clearly, CD47 expression was relatively high in our surgical SCLC cohort. In a recent study, Orozco-Morales et al. evaluated SCLC biopsy specimens and found that 7 out of 29 were positive for CD47.^19^ Although this positivity rate is lower compared to our findings, SCLC may harbor a significant intratumoral heterogeneity, which is highly challenging when assessing expression signatures of various proteins of interest from small biopsy specimens.^12^^,^^31^^,^^32^ Of note, our findings on the relatively high CD47 expression in malignant lung tissue are in line with other studies evaluating CD47 expression in NSCLC. Indeed, Arrieta et al. detected a CD47 positivity of 84% in tumor biopsies with a median expression of 80%.^33^ In a separate study, Zhao et al. found CD47 overexpressed in 65% of the samples when evaluating primary NSCLC specimens; however, their cut-off values were significantly higher compared to our study.^34^ Notably, a further study evaluated the levels of CD47 in NSCLC and pulmonary infectious disease specimens and found significantly higher CD47 expression in malignant than in infectious control tissue.^35^ Accordingly, CD47 represents an immune-checkpoint molecule that is mainly overexpressed by malignant cells. Considering the prognostic value of CD47, we did not identify CD47 as a significant and independent predictor of clinical outcome although higher CD47 expression showed a clear tendency toward shortened OS (31 versus 44 months, *P* = 0.647) and DFS (21 versus 35 months, *P* = 0.165). This trend is consistent with data from other studies showing a correlation between high CD47 expression and worse clinical outcome in several malignancies including acute myeloid leukemia, gastric cancer and ovarian cancer.^36^, ^37^, ^38^ Given the high expression of CD47 in our study cohort and based on the promising findings from preclinical SCLC models underlining the potency of anti-CD47 therapy *in vivo*, further studies are warranted to evaluate CD47 as a promising target in SCLC.^17^

Conversely, we found remarkably lower tumor cell PD-L1 expression than CD47 expression with an overall positivity rate of 9.6% (10/102). Of note, however, other studies reported even lower PD-L1 expression rates in SCLC. Yasuda et al. identified only one PD-L1-expressing tumor from all 39 investigated individuals, referring to an overall positivity rate of 2.5%.^39^ In another study, Schultheis et al. analyzed 94 SCLC patients and none of their specimens showed positive PD-L1 expression in tumor cells.^40^ Importantly, in the current study, we also report that tumoral PD-L1 expression is more frequent among limited-stage (stage I + II) cases compared to advanced-stage patients. Similarly, Fu et al. also reported significantly higher PD-L1 expression in limited-stage SCLC with a positivity rate of 63% among 43 surgically resected patients.^41^ Accordingly, studies evaluating anti-PD-L1 blockage therapies in limited-stage SCLC patients are to be endorsed in the near future.

Similarly to our findings, Schultheis et al. found a higher presence of PD-L1 in tumor stroma although 46% of their investigated samples were small biopsies. Here, tumor stroma can substantially differ from specimens provided by surgical resection.^42^ Nevertheless, 18.5% of the study cohort showed profound PD-L1 expression in tumor-infiltrating lymphocytes (TILs).^40^ Regarding its prognostic value, we found a clear association between stromal PD-L1 presence and favorable OS upon surgical resection for SCLC. Other studies have found the same trends when evaluating ovarian, colorectal or breast cancer patient cohorts as we found in SCLC.^43^, ^44^, ^45^ At first sight, our results that high PD-L1 expression correlates with better oncological outcome might seem counterintuitive. Indeed, PD-L1 is one of the best-described ‘don’t-kill-me’ signals that malignant cells use to suppress inhibition by tumor-specific T cells and thus to evade the physiological antitumor immunity in humans. Accordingly, one might anticipate that high PD-L1 presence automatically associates with worse oncological outcome. However, there are several findings that might serve as a rational explanation for the positive prognostic value of stromal PD-L1 presence. Firstly, Ali et al. described a positive correlation between stromal PD-L1 expression and higher presence of TILs that are known to play a dominant role in the elimination of malignant cells.^46^ Wang et al. also reported on significant PD-L1 up-regulation during the proliferation phase of T cells while Pulko et al. suggested that activated TILs use PD-L1 as a protective signal for their own survival.^47^^,^^48^ Furthermore, up-regulation of PD-L1 signaling has been also shown to be directly promoted by cytokines involved in the antitumor immune response.^49^, ^50^, ^51^ Given these findings, stromal PD-L1 expression might reflect an immunogenic tumor microenvironment that is able to suppress cancer growth and thus explains the association of improved clinical outcome found in our study.^52^^,^^53^

Our group has recently evaluated the clinical relevance of previously presented major molecular SCLC subtypes in a large cohort of surgically resected patients and cell lines.^11^^,^^12^ Beyond prognostic implications, we identified significant associations between distinct molecular subtypes and therapeutically relevant molecules. However, in the current study, neither CD47 nor PD-L1 expression showed an association with any of the major molecular SCLC subtypes.

Notably, our study has limitations that must be acknowledged. Firstly, despite that IHC is a well-established and is a routinely used method for the determination of protein-level alterations in cancer specimens, there are no standardized protocols regarding CD47 and PD-L1 evaluation. Clearly, different antibodies, staining protocols, selected cut-off values and scoring methods could lead to widely differing findings. Secondly, although our study evaluated a comparatively large cohort of surgically resected SCLC cases, the overall number of included patients still remained small. Finally, given its retrospective design, the characterization of the individual survival data has been fully extracted from the available medical records.

### Conclusion

This study aimed to determine the expression pattern, clinicopathological relevance and prognostic importance of CD47 and PD-L1 expression in one of the largest international cohorts of surgically resected SCLC patients to date. We demonstrated that CD47 is highly present in SCLC samples and stromal PD-L1 expression could serve as a prognostic biomarker for a favorable outcome in SCLC. In addition, distinct demographics and therapeutic modalities were identified as relevant factors for optimal survival outcome in surgically managed SCLC patients. Given the need for improved therapeutic concepts in SCLC, further studies are indicated to verify our findings.

## Funding

BD was supported by the Austrian Science Fund [grant numbers FWF I3522, FWF I3977, I4677]. ZM and BD acknowledge funding from the Hungarian National Research, Development and Innovation Office [grant numbers KH130356, KKP126790 to BD; 2020-1.1.6-JÖVő, TKP2021-EGA-33 to BD and ZM]. ZM was supported by the UNKP-20-3 and UNKP-21-3 New National Excellence Program of the Ministry for Innovation and Technology of Hungary, and by the Hungarian Respiratory Society (MPA #2020). VL is a recipient of the Bolyai Research Scholarship of the Hungarian Academy of Sciences and the UNKP-19-4 New National Excellence Program of the Ministry for Innovation and Technology. KS was supported by the Austrian Science Fund (FWF No. T 1062-B33) and the City of Vienna Fund for Innovative Interdisciplinary Cancer Research.

## Disclosure

The authors have declared no conflicts of interest.
