# Supplementary material for: Clinical and prognostic implications of CD47 and PD-L1 expression in surgically resected small-cell lung cancer
Source: ESMO Open. 2022 Nov 16;7(6):100631. doi: 10.1016/j.esmoop.2022.100631 (PMC9808447; doi:10.1016/j.esmoop.2022.100631)
Supplement: Supplementary Table [file mmc1.docx]

**Supplementary Table 1**Clinical and histopathological data of the study population according to CD47 and PD-L1 expressions.

|  | **Total**  **(n=104)** | **CD47^pos^**  **(n=88)** | **CD47^neg^**  **(n=12)** | **t-PD-L1^pos^**  **(n=10)** | **t-PD-L1^neg^**  **(n=92)** | **st-PD-L1^pos^**  **(n=62)** | **st-PD-L1^neg^**  **(n=40)** |
| --- | --- | --- | --- | --- | --- | --- | --- |
| **Age**  (years, median, range) | 63.7  (41.1-83.0) | 63.7  (41.1-83.0) | 64.6 (43.1-74.0) | 65.0 (43.0-71.5) | 63.7 (41.1-83.0) | 64.2 (42.4- 80.5) | 64.0 (41.1-83.0) |
| <65 | 56 (53.8%) | 44 (50.0%) | 8 (66.7%) | 4 (40.0%) | 51 (55.4%) | 31 (50.0%) | 24 (60.0%) |
| ≥65 | 47 (45.2%) | 43 (48.9%) | 4 (33.3%) | 5 (50.0%) | 41 (44.6%) | 30 (48.4%) | 16 (40.0%) |
| *P value* | | *0.2953* | | *0.7282* | | *0.4173* | |
| **Gender** | | | | | | | |
| Female | 49 (47.1%) | 40 (45.5%) | 6 (50.0%) | 3 (30.0%) | 45  (48.9%) | 30  (48.4%) | 18  (45.0%) |
| Male | 54 (51.9%) | 47 (53.4%) | 6 (50.0%) | 6 (60.0%) | 47  (51.1%) | 31  (50.0%) | 22  (55.0%) |
| *P value* | | *0.9999* | | *0.4925* | | *0.6900* | |
| **Smoking status** | | | | | | | |
| Never smoker | 12 (11.5%) | 9 (10.2%) | 2 (16.7%) | 0 | 12 (13.0%) | 6 (9.7%) | 6 (15.0%) |
| Former smoker | 33 (31.7%) | 30 (34.1%) | 3 (25.0%) | 3 (30.0%) | 29  (31.5%) | 20  (32.3%) | 12  (30.0%) |
| Current smoker | 54 (51.9%) | 44 (50.0%) | 7 (58.3%) | 5  (50.0%) | 48  (52.2%) | 33  (53.2%) | 20  (50.0%) |
| N/A | 5 (4.8%) | 5 (5.7%) | 0 | 2 (20.0%) | 3 (3.3%) | 3 (4.8%) | 2 (5.0%) |
| *P value* | | *0.6262* | | *0.5902* | | *0.5302* | |
| **Chronic obstructive pulmonary disease** | 43 (41.3%) | 35 (39.8%) | 7 (58.3%) | 5 (50.0%) | 36 (39.1%) | 27 (43.5%) | 14 (35.0%) |
| *P value* | | *0.3501* | | *0.5177* | | *0.4159* | |
| **Hypertension** | 53 (51.0%) | 49 (55.7%) | 4 (33.3%) | 6 (60.0%) | 46  (50.0%) | 34  (54.8%) | 18  (45.0%) |
| *P value* | | *0.2179* | | *0.7415* | | *0.4178* | |
| **Diabetes** | 17 (16.3%) | 16 (18.2%) | 1 (8.3%) | 2 (20.0%) | 14  (15.2%) | 10  (16.1%) | 6  (15.0%) |
| *P value* | | *0.6851* | | *0.6546* | | *0.9999* | |

|  | **Total**  **(n=104)** | **CD47^pos^**  **(n=88)** | **CD47^neg^**  **(n=12)** | **t-PD-L1^pos^**  **(n=10)** | **t-PD-L1^neg^**  **(n=92)** | **st-PD-L1^pos^**  **(n=62)** | **st-PD-L1^neg^**  **(n=40)** |  |
| --- | --- | --- | --- | --- | --- | --- | --- | --- |
| **Surgery type** | | | | | | | | |
| Sublobar resection | 26 (25.0%) | 22 (25.0%) | 4 (33.3%) | 0 | 25  (27.2%) | 17  (27.4%) | 8  (20.0%) | |
| Wedge resection | 12 (11.5%) | 11 (12.5%) | 1 (8.3%) | 0 | 12  (13.0%) | 9  (14.5%) | 3  (7.5%) | |
| Segmentectomy | 14 (13.5%) | 11 (12.5%) | 3 (25.0%) | 0 | 13  (14.1%) | 8  (12.9%) | 5  (12.5%) | |
| Lobar resection | 64 (61.5%) | 53 (60.2%) | 8 (66.7%) | 7  (70.0%) | 57  (62.0%) | 41  (66.1%) | 23  (57.5%) | |
| Lobectomy | 53 (51.0%) | 43 (48.9%) | 7 (58.3%) | 6  (60.0%) | 47  (51.1%) | 35  (56.5%) | 18  (45.0%) | |
| Pneumonectomy | 11 (10.6%) | 10 (11.4%) | 1 (8.3%) | 1  (10.0%) | 10  (10.9%) | 6  (9.7%) | 5  (12.5%) | |
| Unspecified | 14 (13.5%) | 13 (14.8%) | 0 | 3  (30.0%) | 10  (10.9%) | 4  (6.5%) | 9  (22.5%) | |
| *P value* | | *0.7485* | | *0.1839* | | *0.8078* | | |
| **Molecular Subtype** | | | | | | | | |
| A | 49 (47.1%) | 43 (48.9%) | 4 (33.3%) | 4 (40.0%) | 44  (47.8%) | 31  (50.0%) | 17  (42.5%) | |
| N | 5  (4.8%) | 4  (4.5%) | 1 (8.3%) | 1 (10.0%) | 4  (4.3%) | 3  (4.8%) | 2  (5.0%) | |
| AN | 6 (5.8%) | 6 (6.8%) | 0 | 0 | 6 (6.5%) | 4 (6.5%) | 2 (5.0%) | |
| P | 6 (5.8%) | 5 (5.7%) | 0 | 1 (10.0%) | 5 (5.4%) | 4 (6.5%) | 2 (5.0%) | |
| QNeg | 24 (23.1%) | 17 (19.3%) | 7 (58.3%) | 2 (20.0%) | 22  (23.9%) | 15  (24.2%) | 9  (22.5%) | |
| Unknown | 14 (13.5%) | 13 (14.8%) | 0 | 2 (20.0%) | 11  (12.0%) | 5  (8.1%) | 8  (20.0%) | |
| *P value* | | *0.2110* | | *0.9999* | | *0.9999* | | |
| **Pathologic stage** | | | | | | | | |
| I | 39 (37.5%) | 33 (37.5%) | 5 (41.6%) | 5 (50.0%) | 33  (35.9%) | 27  (43.5%) | 11  (27.5%) | |
| II | 18 (17.3%) | 13 (14.8%) | 3 (25.0%) | 2 (20.0%) | 16  (17.4%) | 8  (12.9%) | 10  (25.0%) | |
| III | 29 (27.9%) | 25 (28.4%) | 4 (33.3%) | 0 | 29  (31.5%) | 20  (32.3%) | 9  (22.5%) | |
| IV | 2 (1.9%) | 2 (2.3%) | 0 | 0 | 2 (2.2%) | 2 (3.2%) | 0 | |
| Unknown | 16 (15.4%) | 15 (17.0%) | 0 | 3 (30.0%) | 12  (13.0%) | 5  (8.1%) | 10  (25.0%) | |
| *P value* | | *0.9999* | | *0.0472** | | *0.4861* | | |

|  | **Total**  **(n=104)** | **CD47^pos^**  **(n=88)** | **CD47^neg^**  **(n=12)** | **t-PD-L1^pos^**  **(n=10)** | **t-PD-L1^neg^**  **(n=92)** | **st-PD-L1^pos^**  **(n=62)** | **st-PD-L1^neg^**  **(n=40)** |  |
| --- | --- | --- | --- | --- | --- | --- | --- | --- |
| **Lymph node status** | | | | | | | | |
| N0 | 44 (42.3%) | 37 (42.0%) | 5 (41.6%) | 6 (60.0%) | 37  (40.2%) | 33  (53.2%) | 10  (25.0%) | |
| N1 | 23 (22.1%) | 19 (21.6%) | 3 (25.0%) | 1  (10.0%) | 22  (23.9%) | 12  (19.4%) | 11  (27.5%) | |
| N2 | 17 (16.3%) | 14 (15.9%) | 3 (25.0%) | 0 | 17  (18.5%) | 10  (16.1%) | 10  (17.5%) | |
| Unknown | 20 (19.2%) | 18 (20.5%) | 1 (8.3%) | 3  (30.0%) | 16  (17.4%) | 7  (11.3%) | 12  (30.0%) | |
| *P value* | | *0.7509* | | *0.1107* | | *0.0240** | | |
| **Tumor size** | | | | | | | | |
| T1 | 42 (40.4%) | 34 (38.6%) | 7 (58.3%) | 3 (30.0%) | 38  (41.3%) | 30  (48.4%) | 11  (27.5%) | |
| T2 | 22 (21.1%) | 17 (19.3%) | 4 (33.3%) | 4 (40.0%) | 18  (19.6%) | 13  (21.0%) | 9  (22.5%) | |
| T3 | 11 (10.6%) | 9 (10.2%) | 1 (8.3%) | 0 | 11  (12.0%) | 5  (8.1%) | 6  (15.0%) | |
| T4 | 12 (11.5%) | 12 (13.6%) | 0 | 0 | 12  (13.0%) | 8  (12.9%) | 4  (10.0%) | |
| Unknown | 17 (16.3%) | 16 (18.2%) | 0 | 3 (30.0%) | 13  (14.1%) | 6  (9.7%) | 10  (25.0%) | |
| *P value* | | *0.5437* | | *0.9999* | | *0.1755* | | |
| **Lesion preoperatively visible during bronchoscopy** | | | | | | | | |
| Yes | 21 (20.2%) | 18 (20.5%) | 3 (25.0%) | 2 (20.0%) | 19  (20.7%) | 10  (16.1%) | 11  (27.5%) | |
| No | 39 (37.5%) | 31 (35.2%) | 7 (58.3%) | 3 (30.0%) | 35  (38.0%) | 27  (43.5%) | 11  (27.5%) | |
| Not performed | 38 (36.5%) | 35 (39.8%) | 1 (8.3%) | 4 (40.0%) | 33  (35.9%) | 20  (32.3%) | 17  (42.5%) | |
| Unknown | 6 (5.8%) | 4 (4.5%) | 1 (8.3%) | 1 (10.0%) | 5  (5.4%) | 5  (8.1%) | 1  (2.5%) | |
| *P value* | | *0.9999* | | *0.9999* | | *0.0958* | | |
| **Chemotherapy** | | | | | | | | |
| Adjuvant | 62 (59.6%) | 50 (56.8%) | 9 (75.0%) | 3  (30.0%) | 58  (63.0%) | 36  (58.1%) | 23  (57.5%) | |
| No adjuvant chemotherapy | 24 (23.1%) | 20 (22.7%) | 2 (16.7%) | 3  (30.0%) | 20  (21.7%) | 12  (19.4%) | 11  (27.5%) | |
| Unknown | 18 (17.3%) | 17 (19.3%) | 1 (8.3%) | 4 (40.0%) | 14  (15.2%) | 12  (19.4%) | 5  (12.5%) | |
| *P value* | | *0.4911* | | *0.3557* | | *0.3051* | | |
